# Constitutive and Induced Expression of Total Phenol and Phenol Oxidases in Wheat Genotypes Ranging in Resistance/Susceptibility to the Root-Lesion Nematode *Pratylenchus thornei*

**DOI:** 10.3390/plants9040485

**Published:** 2020-04-09

**Authors:** Md Motiur Rahaman, Rebecca S. Zwart, John P. Thompson

**Affiliations:** Centre for Crop Health, University of Southern Queensland, Toowoomba, QLD 4350, Australia; MdMotiur.Rahaman@usq.edu.au (M.M.R.); john.thompson@usq.edu.au (J.P.T.)

**Keywords:** *Pratylenchus thornei*, wheat, resistance, defense response, plant phenols, polyphenol oxidase, peroxidase, microplate reader for enzyme assay

## Abstract

Plant-derived phenolic compounds contribute to the defense against various pathogens, including root-lesion nematodes (*Pratylenchus* spp.). However, there are no reports on the role of phenolic compounds in wheat (*Triticum aestivum*) against *Pratylenchus thornei*. In this study, wheat genotypes ranging from resistant to very susceptible to *P. thornei* were used to investigate the level of total phenols and phenol oxidases, polyphenol oxidase (PPO), and peroxidase (POD) expressed in root tissues when grown in the presence and absence of *P. thornei* over time (2–8 weeks). Higher constitutive levels of total phenols were found in resistant synthetic hexaploid wheats CPI133872 (576 µg gallic acid equivalent (GAE)/g root) and CPI133859 (518 µg GAE/g root) at 8 weeks after sowing, compared with moderately resistant and susceptible genotypes (192 to 390 µg GAE/g root). The activity of PPO was induced in resistant (CPI133872) and moderately resistant (GS50a and its derivate QT8343) genotypes, becoming maximal at 4 weeks after *P. thornei* inoculation. The activity of POD was induced in CPI133872 at 6 weeks after *P. thornei* inoculation. Different genetic sources of resistance to *P. thornei* showed diverse defense mechanisms and differences in timing responses. The combined effects of total phenols and oxidative enzymes could be important for defense against *P. thornei* in some resistant wheat genotypes.

## 1. Introduction

The root-lesion nematode *Pratylenchus thornei* is a plant-parasitic nematode that causes yield loss in wheat (*Triticum aestivum*) in many countries [1]. It is one of the major threats to wheat production in the subtropical grain region of eastern Australia [2]. *Pratylenchus* spp. are migratory endoparasites that secrete cell wall degrading enzymes, such as cellulase, glucanase, and pectate lyase, and, together with stylet thrusting and body movement, they penetrate the epidermis of root cells to feed and migrate within the plant root cortex [3]. The feeding, migration, and multiplication of nematodes damages the root system, resulting in the poor uptake of water and nutrients by the plant, which in turn results in yield loss [4,5]. The life cycle (egg, J2, J3, J4, and adult) of *Pratylenchus* is completed within 45 to 60 days [3,6,7], resulting in exponential multiplication of the nematode population densities within the growing season of a susceptible wheat crop [8].

The most effective management strategy for *P. thornei* is the use of resistant wheat cultivars [9,10]. Resistant host plants retard nematode reproduction in roots, reducing the nematode population densities in the soil to attack subsequent crops [9]. No wheat genotype completely prevents the reproduction of *P. thornei*. Hexaploid wheat genotypes (2*n* = 6*x* = 42) with high levels of resistance to *P. thornei* have been identified, including GS50a, a selection from the susceptible wheat cultivar, Gatcher [11], West Asian and North African wheat landraces [12], Iranian wheat landraces [13], and synthetic hexaploid wheat genotypes [10,14,15]. Furthermore, resistance to *P. thornei* has also been identified in tetraploid (2*n* = 4*x* = 28, *Triticum turgidium* subsp. *durum* (AABB genomes) and diploid (2*n* = 2*x* = 14, *Aegilops tauschii* (DD genome) and *Triticum urata* (A^m^A^m^ genome) genome donors of hexaploid wheat [16,17]. Zwart et al. found that the inheritance of resistance in wheat is additive and polygenic [18]. Several quantitative trait loci (QTL) linked to *P. thornei* resistance have been identified in the aforementioned germplasm [14,15,19,20]. However, investigations into the mechanisms of resistance conferred by the quantitative trait loci (QTL) in wheat against *P. thornei* are limited.

The first insights into the biochemical resistance mechanisms in wheat to *P. thornei* suggest that defense responses occur post-penetration of the roots because both resistant and susceptible genotypes were penetrated by approximately similar numbers of nematodes [21]. However, *P. thornei* reproduction rate was found to be significantly less in moderately resistant genotypes than in susceptible genotypes at 16 weeks post-nematode inoculation (PNI) [8]. Thus, upon successful penetration by *P. thornei*, resistance mechanisms of the wheat plant come into play preventing the reproduction and/or feeding of the nematodes. Defense mechanisms activated in the plant root system could include changes in cellular morphology [22] or the production of biochemical compounds such as primary and secondary metabolites, pathogenesis-related proteins such as chitinases, β-1,3 glucanases, peroxidases, and/or lipid transfer proteins [23,24,25]. These changes in defense can be constitutive, such as pre-existing physical barriers or phytoanticipins, or induced phytoalexins that are activated following nematode penetration. Linsell et al. proposed that the defense in resistant wheat against *P. thornei* is constitutive [21]. However, the constitutively expressed biochemical molecules responsible for the defense were not identified in that study.

Phenols are widespread secondary metabolites in plants that have been identified as important compounds in the defense of plants to pathogens [26,27], including defense against certain root-lesion nematode species, namely *P. penetrans, P. coffeae,* and *P. zeae* [28,29,30,31,32]. Phenols are mainly produced through the shikimic acid–phenylpropanoid pathway and range from simple phenols, such as cinnamic acid, to complex phenol polymers such as lignin [33]. Matern and Kneusel proposed that phenol is a rapidly synthesized biomolecule after microbial infection, which can be polymerized by oxidative enzymes into cell walls as lignin [34]. Phenols also help to neutralize reactive oxygen species (ROS) that are produced in plants as an immediate defense against pathogenic attack [35]. The reduction in elevated levels of ROS is important for healthy plant cells after preliminary defense. In addition, the migration of nematodes inside the plant root system can cause root cells to produce free phenols, which react with the plant oxidative enzymes polyphenol oxidase (PPO) and peroxidase (POD) to form toxic quinone [27]. Phenols are also known to be involved in plant pigmentation, growth, signaling molecules, and reproduction [33,34,36].

Increases in phenolic compounds and activity of PPO and POD enzymes have been reported to be both constitutive and induced defense mechanisms in plants against root-lesion nematode infection [35]. Studies on the interaction of banana cultivars with *P. coffeae* showed higher levels of defensive enzymes PPO and POD in both inoculated and non-inoculated treatments of resistant cultivars in comparison to susceptible cultivars, indicating that these enzymes are part of the constitutive defenses of plants [30,36,37]. On the other hand, the amount of the phenolic compound chlorogenic acid and PPO increased significantly in resistant tomato root infected by *P. penetrans*, indicating an induced defense response [38].

There is a lack of knowledge on the role of phenolic compounds and oxidative enzymes PPO and POD in the defense responses in wheat against *P. thornei*. Understanding the constitutive or induced expression of total phenolic compounds in resistant and susceptible wheat genotypes will provide important insights into a possible resistance mechanism of wheat against *P. thornei*. This study focuses on two genetically different sources of *P. thornei* resistance, CPI133872 and GS50a [18], and susceptible wheat genotypes (Janz and Gatcher), and aims to gain insights into possible resistance mechanisms in wheat against *P. thornei* by investigating constitutive and induced levels of (i) total phenols, (ii) PPO activity, and (iii) POD activity, at different time points after nematode inoculation. The relationship between total phenol levels and resistance level is further explored in a larger number of wheat genotypes, ranging from resistant to very susceptible to *P. thornei*.

## 2. Results

### 2.1. Experiment 1: Accumulation of Total Phenols Over Time

**Figure 1:**
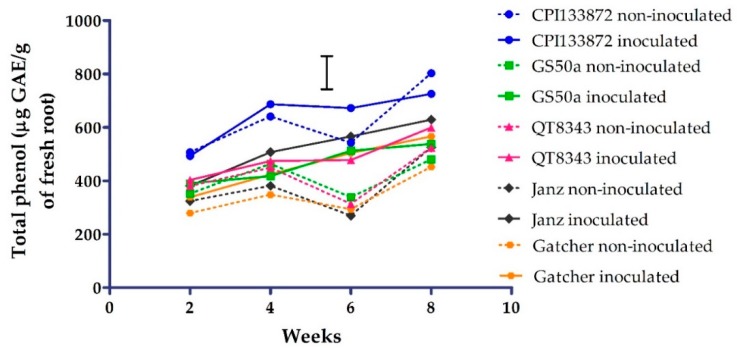
Total phenols (µg gallic acid equivalent (GAE)/g of fresh root) from 2–8 weeks in the roots of *Pratylenchus thornei* inoculated (solid line) and non-inoculated (dotted line) resistant wheat genotypes—CPI133872 (blue), GS50a (green), and QT8343 (red)—and susceptible wheat genotypes—Janz (black) and Gatcher (orange). The values are the means of three replicates. The bar marker indicates the least significant differences (LSD) = 154 (*p* = 0.05) for the interaction genotype* *P. thornei* * time.

The concentration of total phenols in the roots of both resistant and susceptible genotypes increased over time from 2–8 weeks post-nematode inoculation (PNI), with non-inoculated treatments decreasing at 6 weeks before increasing again by 8 weeks (Figure 1). The resistant genotype CPI133872 had significantly higher (*p* < 0.05) concentrations of total phenols than GS50a and QT8343 (a derivate of GS50a) and susceptible genotypes (Janz and Gatcher) for both inoculated and non-inoculated treatments at all time points. The concentration of total phenols for non-inoculated CPI133872 ranged from 506.3 µg gallic acid equivalent (GAE)/g root at 2 weeks to 802.2 µg GAE/g root at 8 weeks. Similarly, the concentration of total phenols for inoculated CPI133872 ranged from 493.0 µg GAE/g root at 2 weeks to 725.4 µg GAE/g root at 8 weeks. Contrastingly, all other genotypes ranged from 279.0–403.4 µg GAE/g root at 2 weeks to 451.8–628.9 µg GAE/g root at 8 weeks.

### 2.2. Experiment 2: Constitutive Levels of Total Phenols in Gnotobiotic Conditions

**Figure 2:**
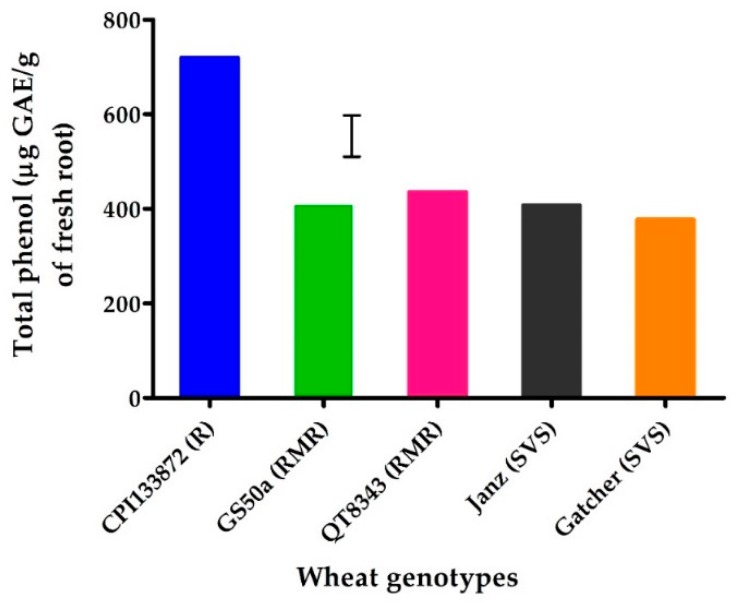
Total phenols (μg gallic acid equivalent (GAE)/g of fresh root) at 3 weeks in the roots of resistant wheat genotypes—CPI133872 (blue), GS50a (green), and QT8343 (red)—and susceptible wheat genotypes—Janz (black) and Gatcher (orange)—grown on agar in gnotobiotic conditions. The values are the means of three replicates. The bar marker indicates LSD = 84 (*p* = 0.05). The *Pratylenchus thornei* resistance rating of each wheat genotype is shown in parenthesis—R = resistant, MR = moderately resistant, S = susceptible, and VS = very susceptible.

The high level of total phenols in non-inoculated resistant genotype CPI133872 was confirmed in an assay conducted under gnotobiotic conditions. Total phenols in non-inoculated treatments for CPI133872 (719 µg GAE/g root) were significantly higher (*p* < 0.05) than all other non-inoculated genotypes, which ranged from 377 µg GAE/g for susceptible genotype Gatcher to 435 µg GAE/g root for moderately resistant genotype QT8343 (Figure 2).

### 2.3. Experiment 3: Total Phenols in Wheat Genotypes Ranging in Resistance/Susceptibility to P. thornei

**Figure 3:**
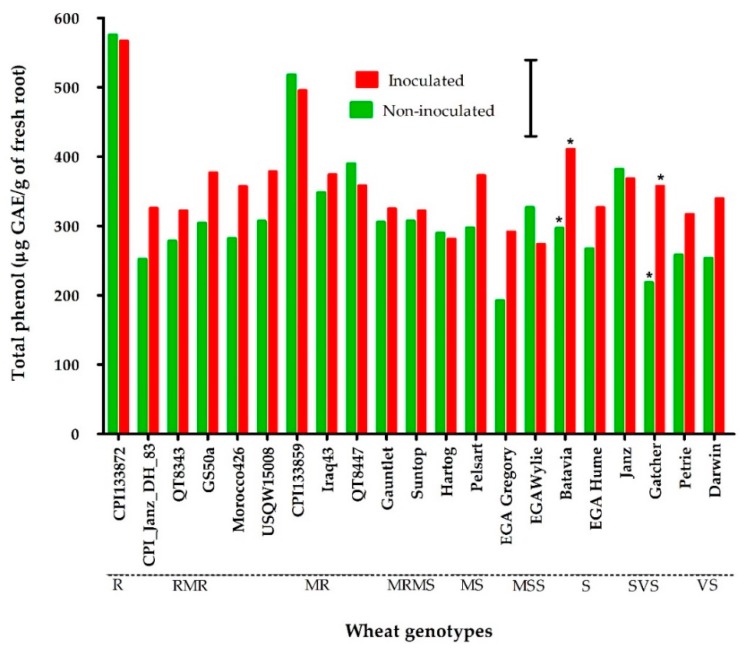
Total phenols (µg gallic acid equivalent (GAE)/g of fresh root) at 8 weeks in the roots of *Pratylenchus thornei* inoculated (red) and non-inoculated (green) wheat genotypes ranging in resistance and susceptibility to *Pratylenchus thornei*. The values are the means of three replications. * represent a statistically significant difference (*p* < 0.05) between inoculated and non-inoculated treatments of a genotype. The bar marker indicates LSD = 110 (*p* = 0.05) for the interaction genotype* *P. thornei*. The resistance rating of each wheat genotype is shown along the X-axis.

The evaluation of the concentration of total phenols in 21 wheat genotypes ranging from resistant to very susceptible at 8 weeks PNI, and including genotypes with several different sources of *P. thornei* resistance, revealed a significantly higher (*p* < 0.05) level of total phenols in the two synthetic hexaploid wheat genotypes, CPI133872 (576 µg GAE/g root) and CPI133859 (518 µg GAE/g root), compared to all other wheat genotypes for both inoculated and non-inoculated treatments (Figure 3).

All other non-inoculated treatments of moderately resistant genotypes, namely GS50a and its derivatives QT8343 and QT8447, landraces originating from the Middle East (Morocco426 and Iraq43), and breeding lines derived from CPI133872 (CPI133872_Janz_DH083 and USQW15008) did not accumulate significantly different levels of total phenols (252–390 µg GAE/g root) than the susceptible genotypes (192–382 µg GAE/g root). The same trend was seen for inoculated treatments, with all sources of resistance (other than CPI133872 and CPI133859) not significantly different (*p* < 0.05) in the level of total phenols than the susceptible genotypes, with levels of total phenols ranging from 274–411 µg GAE/g root (Figure 3). In general, the concentration of total phenols for each genotype did not differ significantly between inoculated and non-inoculated treatments, except for two susceptible genotypes, Gatcher and Batavia, where the inoculated treatment was significantly higher (*p* < 0.05) in total phenols than the non-inoculated treatment: Gatcher (219 µg GAE/g root for non-inoculated and 358 µg GAE/g root for inoculated) and Batavia (297 µg GAE/g root for non-inoculated and 411 µg GAE/g root for inoculated).

### 2.4. Polyphenol Oxidase Enzyme Activity

**Figure 4:**
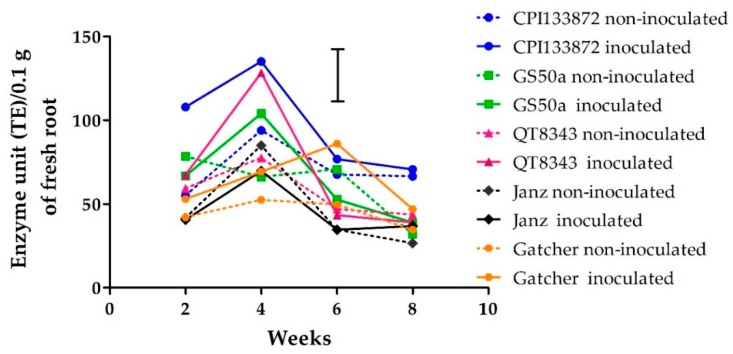
Polyphenol oxidase activity (tyrosinase equivalent (TE)/0.1 g fresh root) from 2–8 weeks in the roots of *Pratylenchus thornei* inoculated (solid line) and non-inoculated (dotted line) resistant wheat genotypes—CPI133872 (blue), GS50a (green), and QT8343 (red)—and susceptible wheat genotypes—Janz (black) and Gatcher (orange). The values are the means of three replicates. The bar marker indicates LSD = 33 (*p* = 0.05) for the interaction genotype* *P. thornei* * time.

Polyphenol oxidase enzyme activity in the roots of resistant and susceptible wheat genotypes varied significantly (*p* < 0.05) over time from 2–8 weeks PNI. At 2 weeks PNI, PPO activity was significantly (*p* < 0.05) higher in only the CPI133872 inoculated treatment (108 tyrosinase equivalent (TE)/0.1 g root) compared with the CPI133872 non-inoculated treatment (55 TE/0.1 g root). For all other genotypes at 2 weeks there were no significant differences (*p* < 0.05) between inoculated and non-inoculated treatments. The activity of PPO increased in inoculated and non-inoculated treatments for all genotypes at 4 weeks and then decreased by 6 weeks and continued to decrease further by 8 weeks (Figure 4). At 4 weeks PNI, the inoculated treatments of the resistant genotype CPI133872 and moderately resistant genotypes GS50a and QT8343 were significantly (*p* < 0.05) higher in PPO activity (103–135 TE/0.1 g root) than the non-inoculated treatments of these genotypes (66–94 TE/0.1 g root). Contrastingly, for susceptible genotypes, no significant differences were found between inoculated (69–85 TE/0.1 g root) and non-inoculated treatments (52–70 TE/0.1 g root). For the susceptible genotype Janz, there were no significant differences between inoculated and non-inoculated treatments at any time point from 2–8 weeks PNI. For the susceptible genotype, Gatcher, the only significant difference (*p* < 0.05) between treatments was recorded at 6 weeks PNI, between inoculated (86 TE/0.1 g root, the highest PPO activity level recorded for this genotype) and non-inoculated treatments (50 TE/ 0.1 g root) (Figure 4).

### 2.5. Peroxidase Enzyme Activity

**Figure 5:**
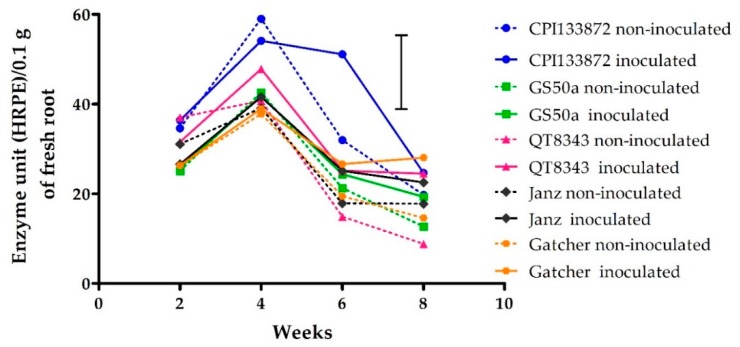
Peroxidase activity (horse radish peroxidase equivalent (HRPE)/0.1 g fresh root) from 2–8 weeks in the roots *Pratylenchus thornei* of inoculated (solid line) and non-inoculated (dotted line) resistant wheat genotypes—CPI133872 (blue), GS50a (green), and QT8343 (red)—and susceptible wheat genotypes—Janz (black) and Gatcher (orange). The values are the mean of three replicates. The bar marker is LSD = 17 (*p* = 0.05) for the interaction genotype* *P. thornei* * time.

Peroxidase enzyme activity varied significantly (*p* < 0.05) over time between inoculated and non-inoculated treatments for only the resistant genotype CPI133872. The activity of POD increased between 2 and 4 weeks for both inoculated and non-inoculated treatments of all genotypes and then decreased at 6 and 8 weeks, for all genotypes except CPI133872 (Figure 5). The highest levels of POD enzyme activity were recorded at 4 weeks for non-inoculated (59 horse radish peroxidase equivalent (HRPE)/0.1 g root) and inoculated treatments (54 HRPE/0.1 g root) of CPI133872. At 6 weeks, the inoculated treatment of CPI133872 maintained high levels of POD activity (51 HRPE/0.1 g root), whereas the non-inoculated treatment of CPI133872 decreased significantly (*p* < 0.05) (31 HRPE/0.1 g root) (Figure 5).

### 2.6. Nematode Quantification

The presence of *P. thornei* inside the root tissue was confirmed in all inoculated treatments by staining and microscopic observation. Nematodes were also extracted at 10 weeks PNI. The total number of *P. thornei*/g of dry root were greatest in Gatcher followed by Janz, QT8343, CPI133872, and GS50a. Similar results were obtained for *P. thornei*/kg of soil and roots (Table 1). All nematode life stages (J2, J3, J4 and adults) were observed present in both the stained root samples and the extracts of soil samples of for all genotypes at 10 weeks PNI.

**Figure 6:**
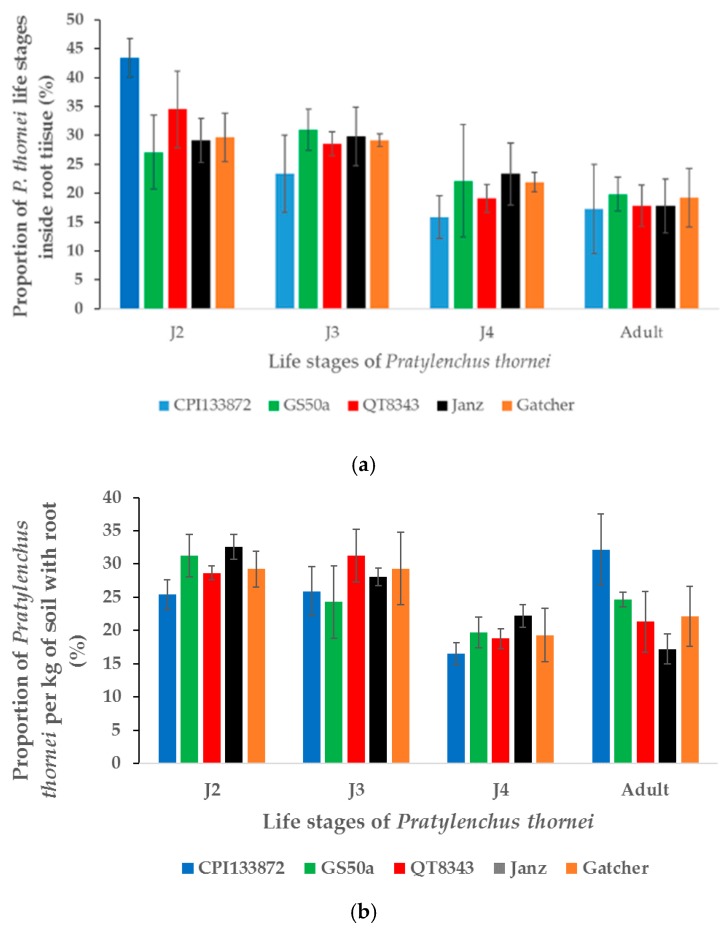
Proportion of *Pratylenchus thornei* life stages (**a**) inside roots of different wheat genotypes and (**b**) per kg of soil and root. The values are the mean ± standard error.

There was no substantial difference in the proportion of life stages among Gatcher, Janz, and QT8343 at 10 weeks PNI. However, some differences in the proportion of life stages per gram of dry root were recorded in Gatcher compared to GS50a and CPI133872 (Figure 6). No contamination of *P. thornei* in non-inoculated treatments was recorded.

### 2.7. Determination of Dry Shoot Weight and Fresh Root Weight

No significant differences (*p* < 0.05) were observed in the mean dry shoot weights (Figure S1) and fresh root weights (Figure S2) of inoculated and non-inoculated treatments for any of the wheat genotypes.

## 3. Discussion

This is the first study to investigate the defensive role of total phenol content and phenol oxidative enzymes (PPO and POD) in wheat against *P. thornei*. In this study, high constitutive total phenol content in the resistant synthetic hexaploid wheat line CPI133872 was confirmed in three independent experiments. Similarly, high constitutive expression of total phenols was found in another resistant synthetic hexaploid wheat line CPI133859. However, moderately resistant genotypes derived from CPI133872, namely CPI133872_Janz_DH83 (a double haploid line from the cross between CPI133872 and Janz) and its derivative USQW15008 (an advanced breeding line from CPI133872_Janz_DH83) did not have higher levels of total phenols than susceptible genotypes at 8 weeks. A similar trend was found in the moderately resistant genotypes GS50a and GS50a-derived lines QT8343 and QT8447, as well as moderately resistant landrace wheats Iraq43 and Morocco426, in which the phenol content was not significantly different from the susceptible genotypes. These results suggest that phenolic compounds may play a role in contributing to *P. thornei* resistance in the synthetic hexaploid lines but not in the other sources of *P. thornei* resistance evaluated in this study.

The synthetic hexaploid wheats CPI133872 and CPI133859 were developed by the International Maize and Wheat Improvement Center (CIMMYT) and share a common durum wheat parent (AABB genome) but different *A. tauschii* parents (DD genome) [10,39]. Studies on the inheritance of *P. thornei* resistance in these two synthetic hexaploid wheats revealed the minimum number of effective genes to be in the range of four to six genes [39], with CPI133872 and CPI133859 sharing at least one common resistance gene [18]. Resistance in GS50a has been determined to be genetically different from that of CPI133872 and CPI133859 [11,18]. High constitutive total phenol content could be an inheritable component of resistance that was not transferred or alternatively was suppressed when CPI133872 was crossed with Janz.

The significant (*p* < 0.05) decline of total phenols in non-inoculated treatments at 6 weeks in Experiment 1 may be due to the changes in plant developmental stage [40]. During this period, wheat is, generally, at the booting and heading stage depending on the genotype [41]. Iannucci et al. also reported that the concentration of phenolic compounds in the rhizosphere soil of wild oat (*Avena fatua*) varied with the age of the plant [42]. A sharp decline in phenolic compounds has been reported in that study during stem elongation and early booting stage [42]. However, the total phenol content at 6 weeks PNI in inoculated treatments for both resistant and susceptible genotypes did not decline, contrary to the non-inoculated treatments. This suggests that *P. thornei* infestation might induce changes in phenylpropanoid pathways to keep the phenol production at an increased level irrespective of the changes in the developmental stage of wheat genotypes. Rahman et al. recently identified key enzymes of phenylpropanoids, such as phenyl ammonium lyase and chalcone synthase, as candidate genes in QTL regions on chromosomes 2BS and 6DS for resistance to *P. thornei* in the synthetic derived wheat line Sokoll [43]. Initial QTL mapping studies reported the co-location of resistance to *P. thornei* in Sokoll on chromosomes 2BS and 6DS with QTL for resistance to *P. thornei* in CPI133872 [14,19]. However, more recent mapping studies have identified kompetitive allele specific PCR (KASP) markers that select for the CPI133872-derived resistance, but do not select for Sokoll-derived resistance, suggesting that *P. thornei* resistance in these synthetic hexaploid wheat genotypes is controlled by different genes [44] and therefore with possibly different resistance mechanisms.

Both constitutive and induced phenolic compounds have been previously reported to be responsible for defense in host plants against various root-lesion nematodes [35,36,37,38]. Backiyarani et al. reported that total phenol in non-inoculated resistant banana genotypes was higher than in the non-inoculated susceptible genotype, suggesting potential constitutive biochemical defensive against *P. coffeae* infestation [30]. Constitutive phenols were also responsible for the inhibition of burrowing nematode *Radopholus similis* infection in banana cultivars [45]. The amount of the phenolic compound chlorogenic acid and PPO increased significantly in the roots of resistant tomato infected by *P. penetrans*, indicating an induced defense response [46].

Increases in the levels of phenol oxidative enzymes in plants wounded and/or infected by pathogens is well documented. As key enzymes acting on the phenylpropanoid pathway, PPO and POD could play important roles in plant resistance to nematodes in several ways; (a) reaction with plant phenolics to form toxic quinone, (b) the production of reactive oxygen species that can act on pathogens or initiate defensive gene expression, (c) reduction in the availability of cellular protein to plant pathogens, (d) crosslinking phenolic compounds into lignin and other cell wall polymers to strengthen cell walls as a physical barrier to pathogens, and possible formation of brown melanin polymer crosslinking phenols in presence of cellular proteins and amino acids [47,48,49,50]. In our study, levels of PPO and POD were higher in CPI133872, GS50a, and QT8343 at 4 weeks PNI than in the susceptible genotypes Janz and Gatcher. Combined effects of oxidative enzymes and total phenols at 2 and 4 weeks PNI could be a key factor for defense in *P. thornei* resistant and moderately resistant wheat genotypes. Higher levels of phenols, PPO and POD, at 2 and 4 weeks PNI could be a factor contributing to the superior resistance of CPI133872 to *P. thornei*.

Comparative enzyme assays of PPO and POD in the roots of different wheat genotypes were performed using a microplate reader to measure absorbance. Microplate readers have not been commonly used previously for enzyme assays of plant extracts [51]. The concentration of substrates and enzyme extracts was optimized to obtain results in the linear range of enzyme kinetics, and the results were expressed according to standard enzyme activities instead of the international unit (IU) of enzyme activities. A major benefit of using a microplate reader in comparison to cuvette assays is that multiple samples can be analyzed at the same time, which is required for analyzing plant samples with a high number of treatments and biological replicates. Moreover, due to the speed and reproducibility of the procedure, multiple assays can be performed under the same controlled conditions.

Resistance in wheat against *P. thornei* occurs post nematode penetration of the wheat root [21,52]. Significant differences in *P. thornei* numbers between resistant and susceptible wheat genotypes can be reliably found at later times (16 weeks PNI) of plant growth, in which time several life cycles of the nematodes contribute to the exponential increase in nematode numbers [8]. The time points and the tissue location of total phenolic compounds could be very important factors for providing defense against *P. thornei*. Lignin biosynthesis is linked with phenylpropanoid metabolism, which leads to total phenol expression [53]. Higher contents of the oxidative enzymes polyphenol oxidase and peroxidase in moderately resistant genotypes at 2–4 weeks PNI could enhance the polymerization of the phenol monomer monolignol to form lignin [29,54]. The conversion of monolignol into lignin not only can give rigidity to cell walls to retard nematode movement and penetration but it can also protect plant cellulose from nematode degradation [55]. Increases in the total phenols and lignin content of plant cell walls were identified as induced defense responses in resistant banana cultivars on infection by the burrowing nematode *Radopholus similis* [56]. The estimation of lignin in roots of different wheat genotypes is required to provide insights into the relationship between total phenols and lignin content over time in *P. thornei* resistant wheat genotypes.

The rapid accumulation of phenolic compounds at the infection sites of plants is a well-documented initial defense response of plants to pathogen infection. Increase in total phenolic compounds at infection sites in susceptible genotypes could be a part of a hypersensitive reaction rather than providing defense in other uninfected parts of the roots. Relatively lower PPO and POD activity in susceptible wheat genotypes might also be responsible for the ineffectiveness of the increased total phenol as a defense mechanism in susceptible genotypes. Hung et al. proposed that oxidized phenol potentially acts against the root-knot and root-lesion nematodes, not the phenol itself [46].

Nematode extraction and root staining confirmed that there was infection in the wheat roots in all inoculated samples. The numbers of *P. thornei* were significantly greater in susceptible genotypes (Gatcher and Janz) than in the resistant (CPI133872) and moderately resistant (GS50a, QT8343) genotypes when extracted at 10 weeks. The compound effect of nematode reproduction at different rates inside different wheat genotypes magnify differences at 16 weeks of plant growth [8]. The *P. thornei* inoculum used in the experiments contained mixed life stages in approximately equal proportions. Interestingly, soil plus roots from the resistant genotype CPI133872 contained a higher proportion of adult *P. thornei* than soil from other genotypes at 10 weeks PNI. Flavonoids have been shown to reduce the hatching of nematode eggs [57], suggesting that phenolic compounds could affect the proportion of nematode life stages. Furthermore, isoflavonoids are phenolic compounds that have been causally linked to the reduced motility of *P. scribneri* in resistant lima beans [58] and *P. penetrans* in lucerne [38]. The resistance of CPI133872 could result in overall fewer numbers of new juveniles, or the juveniles and adults could leave the roots due to unfavorable conditions inside resistant root tissues. Further detailed histopathology observations of the *P. thornei*–wheat interaction over time would be valuable in understanding the effect of host resistance on the nematode life stages.

Knowledge of the specific types of phenolic compounds and other secondary metabolites in resistant and susceptible genotypes at specific times of *P. thornei* infestation in future studies could further improve our understanding of the defense mechanisms. Moreover, further investigations into larger numbers of wheat lines derived from the synthetic hexaploids CPI133872 and CPI133859 would evaluate the predictive value of total phenol, or specific phenolic compounds, as well as phenol oxidative enzymes, as metabolic biomarkers [59,60] for predicting *P. thornei* resistance phenotypes. A holistic metabolomics approach is recommended for future studies.

## 4. Materials and Methods

### 4.1. Plant Material and Growth Conditions

#### 4.1.1. Experiment 1: The Accumulation of Total Phenols and Oxidative Enzymes (PPO and POD) Over Time

Two susceptible wheat genotypes (Gatcher and Janz) and three resistant to moderately resistant wheat genotypes (CPI133872, GS50a, and QT8343) were grown as a glasshouse experiment at the Leslie Research Facility (27°32′02″ S 151°56′09″ E), Toowoomba, Australia, in August to October. Treatments were replicated three times in a factorial randomized block design with five genotypes, two nematode treatments (inoculated and non-inoculated), and five harvest time points (2, 4, 6, 8, and 10 weeks post-nematode inoculation (PNI)).

A self-mulching black vertisolic soil was collected from Formartin, Australia (27°22′12″ S 151°25′53″ E), and pasteurized in an air: steam stream at 85 °C for 45 min [61]. The soil moisture content was determined by drying 100 g of steam-pasteurized soil at 105 °C for 48 h. Slow-release fertilizer Osmocote^®^ Plus Trace Elements (Scott Australia, Baulkham Hills, Australia) was used at the rate of 1 g per pot of 330 g soil (oven-dried equivalent). The pots were placed on glasshouse benches fitted with a continuous bottom watering system with water tension maintained at 2 cm by a capillary system using bidim^®^ matting (Geofabrics Australasia, Brisbane, Australia) [62]. The inoculated treatments were separated from the non-inoculated treatments by a 5-cm gap between the capillary mats to avoid cross-contamination. Under-bench heating was used to maintain soil temperature at 22 ± 4 °C, the optimum temperature for *P. thornei* reproduction [63]. Soil temperature was monitored throughout the experiment using iButtons (Thermochron^®^, Baulkham Hills, Australia) placed in arbitrarily selected pots at 3 cm of depth in the soil.

Two seeds were sown on a base layer of soil (80% of the total soil volume). Nematodes were added in a 10-mL liquid suspension to inoculated treatments, at the rate of 3300 *P. thornei* (J2: 23.5%, J3: 30%, J4: 16.5% adult 30%) equivalent to 10 nematodes/g of oven-dried soil per pot (70 mm square, 150 mm height). The seed and nematodes were covered with the remaining 20% soil [62]. Ten days after sowing, plants were thinned to one seedling per pot with a scalpel blade, retaining roots inside the pots. The roots of each genotype were assessed for total phenol content and oxidative enzymes (PPO and POD) at 2, 4, 6, and 8 weeks PNI. A final time-point at 10 weeks was used to assess nematode reproduction in the roots and soil for each genotype.

#### 4.1.2. Experiment 2: Constitutive Levels of Total Phenols in Wheat Roots under Gnotobiotic Conditions

Wheat seeds of five genotypes (Gatcher, Janz, GS50a, QT8343 and CPI133872), were grown without *P. thornei* inoculation in a controlled environment growth cabinet (Bioline, Percival Scientific, IA, USA) for 3 weeks. The seeds were surface sterilized according to Wu et al. with slight modification [64]. The seeds were covered in 70% ethanol for 5 min followed by diluted bleach solution (NaOCl) (2.5%) for 15 min. The seeds were then rinsed with sterile Milli–Q^®^ (Merck, Darmstadt, Germany) reverse osmosis water five times. The seeds were imbibed by soaking in sterile Milli–Q^®^ water for 12 h, then placed on autoclaved petri plates containing moistened sterile filter paper and allowed to germinate for 48 h. Two-day old surface-sterilized and pre-germinated seedlings were transferred aseptically into Schott bottles (DURAN^®^ GLS 80, DWK Life Sciences, Wertheim, Germany) containing 200 mL of 0.3% autoclaved tap water agar [64]. Four seedlings were placed on the agar per bottle. The lids were screwed on loosely and then wrapped with parafilm. The experiment was arranged in a completely randomized design with three replicate bottles for each genotype.

Light intensity inside the growth cabinet was maintained at 400 µ mole/m^2^ with a photoperiod of 13 h light and 11 h dark. The intensity of the light was measured with a photosynthetically active radiation (PAR) light meter (Li-250A, Li-COR Bio-Sciences, Lincoln, NE, USA). The light wavelength was in the PAR range of 400 to 700 nm. The temperature was maintained at 22 ± 2 °C throughout the experiment.

#### 4.1.3. Experiment 3: Total Phenols in Wheat Genotypes Ranging in Resistance/Susceptibility to *P.* *thornei*

Twenty-one wheat genotypes were selected to cover a range of resistance/susceptibility to *P. thornei* [65] (Table 2). The wheat genotypes were grown using the same glasshouse conditions as described for Experiment 1. Treatments were replicated three times in a split plot design with plus or minus *P. thornei* inoculation randomized to the main plots and wheat genotypes randomized in the sub-plots in a row : column design. The plants were grown in the glasshouse for 8 weeks from July to September.

### 4.2. Sample Collection and Storage

The plant roots from the pot experiments were washed under running tap water to remove the soil, taking care to minimize damage to the roots. The roots were then washed with sterile Milli–Q^®^ (Merck, Darmstadt, Germany) reverse osmosis water and then blotted dry with tissue paper. The plant roots from the Schott bottles were removed from the agar and blotted dry gently with tissue paper. The fresh root weight of each plant was recorded. Small pieces (4 to 6 cm) of the roots (300 mg) from Experiments 1 and 3 were sampled for acid fuchsin staining, to check nematode infection in the roots of the collected samples. The remaining roots were placed inside 50-mL screwcap falcon tubes and immediately frozen with liquid nitrogen. Frozen roots were stored at −80 °C until analyses for total phenols and oxidative enzymes were undertaken. Whole root and soil from the last time point (10 weeks) from Experiment 1 was stored at 4 °C and later used for nematode extraction. The plant tops were oven dried for 48 h at 80 °C to determine dry weight.

### 4.3. Estimation of Total Phenols

The total phenol content of the roots was determined by the Folin–Ciocalteu method [66] with slight modifications. A sample of the root material (0.1 g fresh weight) from each treatment, previously ground and homogenized in liquid nitrogen with a chilled mortar and pestle, was transferred to a fresh microcentrifuge tube. Two milliliters of 95% methanol was added to the finely ground root powder. The root powder in methanol was transferred into a 2 mL microcentrifuge tube and incubated at room temperature for 48 h. The samples were then centrifuged (Centrifuge 5424 R, Eppendorf, Hamburg, Germany) at 13,000 g for 5 min and the supernatant containing the root extract was transferred to a fresh microcentrifuge tube. One hundred microliters of root extract was mixed with 200 µL of 0.2 N Folin–Ciocalteu reagents, a mixture of phosphomolybdate and phosphotungstate (Sigma Aldrich, St Louis, MO, USA). Blank tubes were prepared using 100 µL of 95% methanol in place of the root extract. The reaction tubes were thoroughly mixed by vortexing and then incubated for 5 min at room temperature. Eight hundred microliters of sodium carbonate solution (700 mM) was added to the reaction mixture and further incubated for 2 h at room temperature in the dark. The tubes were centrifuged at 3000 g for 30 s to remove any suspended particles. Two hundred microliters of the supernatant from each sample were transferred to a 96-well microplate (Costar^®^, Thermo Fisher Scientific, Waltham, MA, USA). The absorbance was measured at 765 nm using a microplate reader (Fluostar Omega, BMG Labtech, Mornington, Australia). The total phenol content was determined by comparison with a standard curve for gallic acid (Sigma Aldrich, St Louis, MO, USA) in the range 0 to 200 µg/mL (Figure S3). The samples were prepared in triplicate and the best-fitted results were used for the calculation of total phenols in the root samples under the same conditions and using the same stock of reagents. Three technical replicates for each of three biological replicates were analyzed and mean values expressed as µg of gallic acid equivalent (GAE)/g of fresh root.

### 4.4. Preparation of Enzyme Extracts and Optimization of Protocol for Microplate Reader

Assays for PPO and POD were performed with root materials collected from Experiment 1. The concentrations of substrates for both PPO and POD were optimized prior to performing the enzyme assays with the wheat root extracts. Enzyme kinetics were tested with commercially purchased tyrosinase (Sigma Aldrich, St Louis, MO, USA, ≥1000 unit/mg of solid) with pyrocatechol as substrate for PPO activity (Figure S4) and horseradish peroxidase (Sigma Aldrich, St Louis, MO, USA, ≥250 unit/mg of solid) with guaicol as substrate in the presence of hydrogen peroxide for POD activity (Figure S5) to prepare standard curves. A regression equation in the linear range from the standard curve was used to calculate enzyme activity in the wheat root tissue samples [67]. Different concentrations of wheat root extracts were tested to find the linear range within the value of standard curve. The amount of PPO in wheat root extracts was expressed as tyrosinse equivalent (TE)/0.1 g fresh root. The amount of POD was expressed as horseradish peroxidase equivalent (HRPE)/0.1 g fresh root, respectively.

A microplate reader was used to test multiple samples at a time according to Siguemoto and Gut [51] with modification. The protocol was optimized for a microplate reader to calculate PPO activities in multiple wheat samples at a time. Enzymes were extracted from 100 mg of root powder in 0.05 M sodium phosphate buffer (NaH_2_PO_4_ and Na_2_HPO_4_) at pH 7. Root materials were ground in liquid nitrogen with a chilled mortar and pestle. The root powder was homogenized with 2 mL of phosphate buffer and kept at 4 °C overnight. The homogenate solution was centrifuged at 15,000 rpm for 15 min at 4 °C [68]. The supernatants were transferred to fresh 2 mL microcentrifuge tubes and kept at 4 °C until used for the enzyme assay. One hundred microliter of the supernatant was diluted to 1 mL with phosphate buffer. This supernatant was used as the enzyme extract. Enzyme assays were completed within 2 h of extraction. A multichannel pipette was used to pipette buffer and enzyme extracts followed by the respective substrates of both PPO and POD assays. The assays were done in 96 well microplates (Costar^®^, Thermo Fisher Scientific, Waltham, MA, USA).

### 4.5. Polyphenol Oxidase Enzyme Assay

For the PPO enzyme assay, 33 µL of the enzyme extract was added to 100 µL of sodium phosphate buffer (pH 7). The solution mixture was kept at 25 °C for 15 min to equilibrate the temperature. Freshly prepared 0.05 M pyrocatechol (Sigma Aldrich, St Louis, MO, USA) solution was also left to equilibrate at 25 °C for 15 min. After equilibration, 67 µL of pyrocatechol was added to the buffer and the enzyme. The absorbance was taken every 30 s for 3 min at 420 nm wavelength using a microplate reader (Fluostar Omega, BMG Labtech, Mornington, Australia). A blank was set with phosphate buffer and pyrocatechol without the addition of enzymes. The mean enzyme activity for each root sample was calculated from absorbance values at 30 and 60 s using the regression equations relating enzyme activity to absorbance at those respective times (Figure S4).

### 4.6. Peroxidase Enzyme Assay

The peroxidase enzyme assay was performed with the same enzyme extracts as used for the PPO assay. Forty-two microliters of enzyme extract was added to 125 µL of buffer. The solutions were kept at 25 °C for 15 min to equilibrate along with freshly prepared 10 mM guiaiacol (Sigma-Aldrich, St Louis, MO, USA) and 6.4 mM hydrogen peroxide (Sigma Aldrich, St Louis, MO, USA). Then, 17 µL of equilibrated guaiacol was added to the buffer enzyme mixture followed by 17 µL of hydrogen peroxide. The absorbance of the resultant mixture was recorded at 470 nm every 30 s for 3 min. The blank was prepared with phosphate buffer and pyrocatechol without the addition of enzymes. The absorbance values at 30 and 60 s were used for the calculation of enzyme activity as per the regression equation of the standard curve at those respective times (Figure S5).

### 4.7. Nematode Quantification

*Pratylenchus thornei* were extracted from the roots and soil of the samples collected at the 10-week time point in Experiment 1, using the Whitehead tray method [69]. The roots were separated from soil, washed, and chopped into smaller pieces (1 to 3 cm) and placed on a Whitehead tray for extraction at 22 °C. The remainder of the soil samples were put on another Whitehead tray separately to the roots. The root and soil samples were incubated at 22 °C with 1 L water per tray for 7 d and 4 d, respectively, to allow the nematodes to migrate from the roots and soil into the water. A 20-µm aperture sieve was used to collect the nematodes in 28-mL sample tubes. Following nematode extraction, the root samples were oven dried at 80 °C for 48 h and the dry weight of the roots was recorded. The extracted nematodes were counted using a 1-mL Peters nematode counting chamber (Chalex Corporation, Park City, USA) under an Olympus BX53 compound microscope (Olympus, Tokyo, Japan). The nematode numbers were expressed per g of dry root and per kg of soil, respectively.

Root samples from Experiments 1 and 2 were stained with 0.1% acid fuchsin according to a method modified from Bybd et al. [70]. The roots were chopped and placed inside a stain tube [71] and 10 mL of acid fuchsin stain (0.1% *w*/*v*, prepared in 90% lactic acid solution) were added to each tube. The roots in acid fuchsin solution were heated in a boiling water bath for 2 min, washed in tap water, and then transferred to a sample tube containing 90% lactic acid solution. Three drops of 8 N HCl were added per tube to aid destaining of the root tissues. The stained nematodes in the roots were observed under an Olympus BX53 compound microscope (Olympus, Tokyo, Japan) in both bright field and differential interference contrast (DIC) modes.

### 4.8. Statistical Analysis

Experimental designs and data analysis were performed using R-software version 3.5.1 [72] and Genstat^®^ for Windows™ [73]. Analysis of variance (ANOVA) was conducted for total phenol content, PPO activity, and POD activity. The significance of the differences in total phenols, PPO activity, and POD activity was tested among genotypes and the inoculation treatments at a 5% significance level using the least significant differences (LSD).

## 5. Conclusions

Our study has revealed high constitutive levels of total phenols in the synthetic hexaploids CPI133872 and CPI133859. The activity of PPO was induced in resistant (CPI133872) and moderately resistant (GS50a and derivate QT8343) genotypes and was maximal at 4 weeks after *P. thornei* inoculation. The activity of POD was induced in CPI133872 at 6 weeks after *P. thornei* inoculation. Different genetic sources of resistance to *P. thornei* showed diverse defense mechanisms and differences in the timing of responses. The combined effects of total phenols and oxidative enzymes could be important for defense against *P. thornei* in some resistant wheat genotypes.

## Figures and Tables

**Table 1:** Total *Pratylenchus thornei* numbers (natural log transformed mean value) in different wheat genotypes at 10 weeks post-nematode inoculation.

Genotype	ln (*P. thornei*/g of Dry Root + 1) (Back Transformed Mean))	ln (*P. thornei*/kg of Soil and Root + 1) (Back Transformed Mean)
CPI133872	7.859 ^b^ (2588)	8.295 ^b^ (4004)
GS50a	7.783 ^b^ (2399)	8.536 ^b^ (5095)
QT8343	8.074 ^ab^ (3210)	8.733 ^b^ (6204)
Janz	8.472 ^ab^ (4779)	9.285 ^ab^ (10775)
Gatcher	8.997 ^a^ (8079)	10.018 ^a^ (22426)

Means followed by the same letter in a column are not significantly different by LSD (*p* < 0.05).

**Table 2:** Origin and resistance rating to *Pratylenchus thornei* for wheat genotypes.

Genotype	Origin	Rating [65]
CPI133872	Synthetic hexaploid	R
CPI133872_Janz DH083	CPI133872 derived line	R-MR
QT8343	GS50a derived line	R-MR
GS50a	Selection from Gatcher	R-MR
Morocco 426	Landrace	R-MR
USQW15008	CPI133872_Janz_DH083 derived line	MR
CPI133859	Synthetic hexaploid	MR
Iraq 43	Landrace	MR
QT8447	GS50a derived line	MR
Gauntlet	Cultivar	MR-MS
Suntop	Cultivar	MR-MS
Hartog	Cultivar	MS
Pelsart	Cultivar	MS
Gregory	Cultivar	MS-S
Wylie	Cultivar	MS-S
Batavia	Cultivar	S
Hume	Cultivar	S
Janz	Cultivar	S-VS
Gatcher	Cultivar	S-VS
Petrie	Cultivar	VS
Darwin	Cultivar	VS

R = resistant, MR = moderately resistant, MS = moderately susceptible, S = susceptible, VS = very susceptible.

## Supplementary Materials

The following are available online at https://www.mdpi.com/2223-7747/9/4/485/s1, Figure S1: Dry shoot weight (g) of *Pratylenchus thornei* inoculated and non-inoculated treatments of five wheat cultivars over the time points (2–10 weeks) Figure S2. Fresh root weight of inoculated and non-inoculated treatment of five wheat genotypes over the time points (2–8 weeks). Figure S3. Standard curve of gallic acid for total phenols estimation in wheat root samples. Figure S4 and Figure S5: Standard curves of tyrosinase and horseradish peroxidase in linear range of concentration at different time (30–180 s) of enzyme assay progression.
