# Supplementary material for: Constitutive and Induced Expression of Total Phenol and Phenol Oxidases in Wheat Genotypes Ranging in Resistance/Susceptibility to the Root-Lesion Nematode Pratylenchus thornei
Source: Plants (Basel). 2020 Apr 9;9(4):485. doi: 10.3390/plants9040485 (PMC7238097; doi:10.3390/plants9040485)
Supplement: Supplementary file 1 [file plants-09-00485-s001.pdf]

plants

MDPI

Supplementary Material

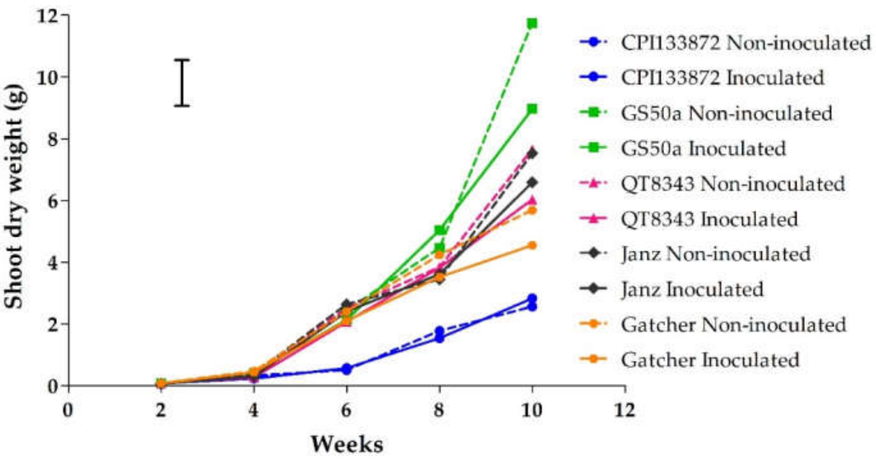

Figure S1. Dry shoot weight (g) of *Pratylenchus thornei* inoculated and non-inoculated treatments of five wheat cultivars over the time points (2–10 weeks). Values are the mean of three replicates. Bar marker indicates LSD = 1.72 (p = 0.05), for the interaction genotype* *P. thornei* * time.

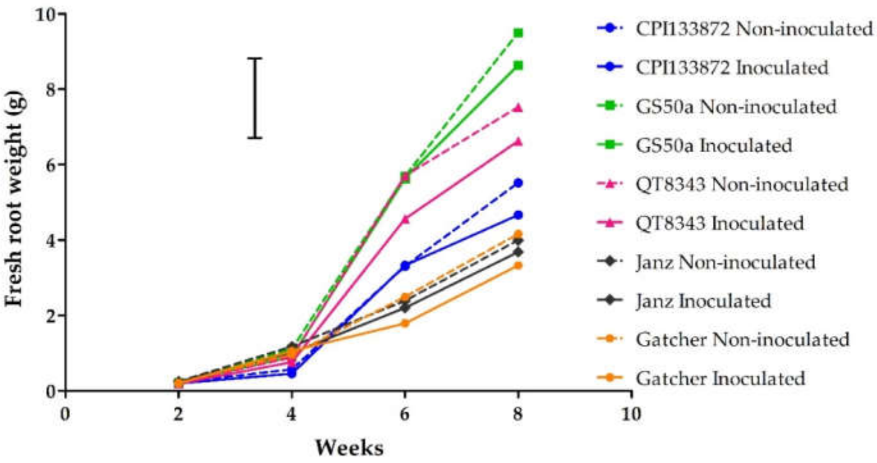

Figure S2. Fresh root weight of inoculated and non-inoculated treatment of five wheat genotypes over the time points (2–8 weeks). Values are the mean of three replicates. Bar marker indicates the least significant differences LSD =2.17 (p = 0.05), for the interaction genotype* *P. thornei* * time.

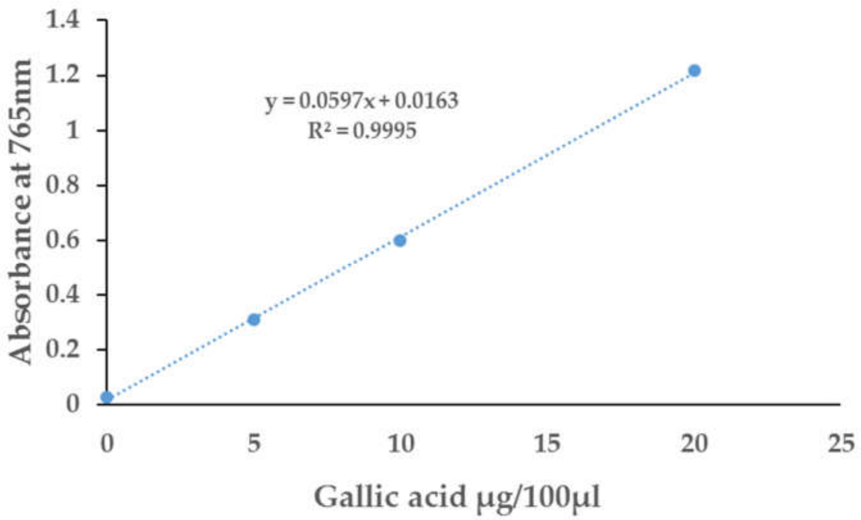

Figure S3. Standard curve of gallic acid for total phenols estimation in wheat root samples.

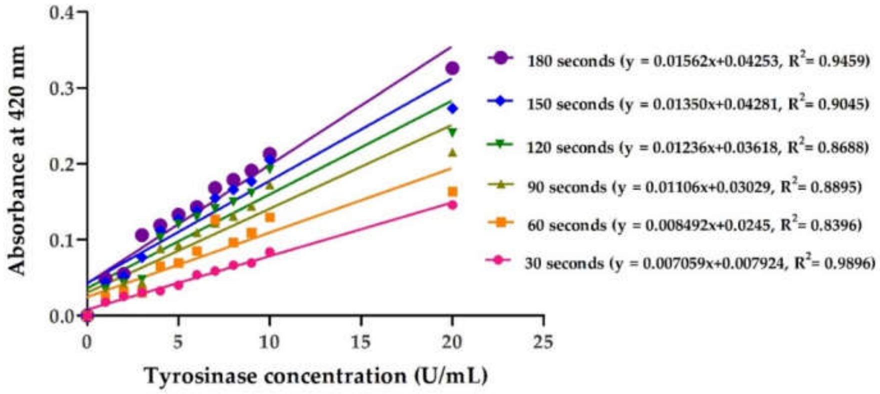

Figure S4. Standard curve of tyrosinase in linear range of concentration at different times (30–180 Seconds) of the enzyme assay progression reacting with pyrocatechol (50 mM) substrate.

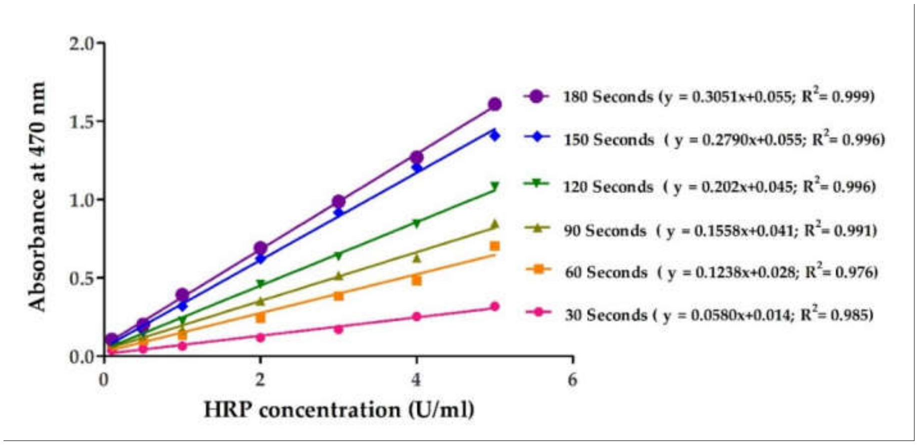

Figure S5. Standard curve of horseradish peroxidase (HRP) in linear range of concentration at different times (30–180 seconds) of the enzyme assay progression reacting with guaiacol (10 mM) and hydrogen peroxide (6.4 mM) substrate.
